# Employment Status, Housing Stability, and Cognitive Health: Socioeconomic Determinants of Alzheimer's Disease and Dementia in Western and Eastern Africa

**DOI:** 10.1002/alz70858_107612

**Published:** 2025-12-25

**Authors:** Carolina Scaramutti, Dingtian Cai, Farid Rajabli, Larry D Adams, Kara L Hamilton‐Nelson, Michael L Cuccaro, Daniel A. Dorfsman, Joshua O Akinyemi, Kyle M Scott, Motunrayo Coker, Kazeem Akinwande, Samuel Diala, Patrice G Whitehead, Mayowa Ogunronbi, Albertino Damasceno, Andrew F Zaman, Biniyam A Ayele, Allison M Caban‐Holt, David Ndetei, Fred Stephen Sarfo, Susan H. Blanton, Albert Akpalu, African Dementia Consortium, Alzheimer's Disease Sequencing Project (ADSP), Margaret Pericak‐Vance, Azizi A Seixas

**Affiliations:** ^1^ Department of Psychiatry and Behavioral Sciences, University of Miami Miller School of Medicine, Miami, FL, USA; ^2^ John P. Hussman Institute for Human Genomics, University of Miami Miller School of Medicine, Miami, FL, USA; ^3^ Dr. John T. Macdonald Foundation Department of Human Genetics, University of Miami Miller School of Medicine, Miami, FL, USA; ^4^ University of Miami Miller School of Medicine, Miami, FL, USA; ^5^ University of Miami, Miami, FL, USA; ^6^ Dr. John T. MacDonald Foundation, Department of Human Genetics, University of Miami, Miami, FL, USA; ^7^ College of Medicine, University of Ibadan, Ibadan, Oyo, Nigeria; ^8^ Neuroscience And Ageing Research Unit, Institute for Advanced Medical Research and Training, College of Medicine, University of Ibadan, Ibadan, Nigeria; ^9^ Cell Biology and Genetics Unit, Department of Zoology, University of Ibadan, Ibadan, Nigeria; ^10^ Faculty of Medicine, Eduardo Mondlane University, Maputo, Mozambique; ^11^ Global Brain Health Institute, San Francisco, CA, USA; ^12^ Wake Forest University School of Medicine, Winston‐Salem, NC, USA; ^13^ Maya Angelou Center for Health Equity, Wake Forest School of Medicine, Winston‐Salem, NC, USA; ^14^ Kwame Nkrumah University of Science and Technology, Kumasi, Ghana, Kumasi, Ghana; ^15^ University of Ghana Medical School, Accra, Ghana; ^16^ University of Pennsylvania, Philadelphia, PA, USA; ^17^ John P. Hussman Institute for Human Genomics, Miller School of Medicine, Miami, FL, USA

## Abstract

**Background:**

Socioeconomic factors, including employment status and housing stability, are critical determinants of health outcomes. Economic hardship increases health risks by limiting healthcare access, exacerbating stress, and contributing to poorer mental and physical health. Employment status influences access to healthcare and financial security, while housing instability is linked to psychological distress and chronic disease. This study examines the association between cognitive health diagnoses (Non‐Cognitively Impaired, MCI, AD, Dementia, Other or No Diagnosis) and key socioeconomic factors, including employment and housing stability.

**Method:**

A quantitative analysis was conducted using two regional samples from the U19 READ‐ADSP DAWN Study: Western Africa (*n* = 598) and Eastern Africa (*n* = 631). Assessed variables included employment status (full‐time, part‐time, retired, unemployed seeking/not seeking work), unemployment benefits/government assistance (Yes/No), housing security (worry about losing housing: Yes/No), and cognitive health diagnosis (Non‐Cognitively Impaired, MCI, AD, Dementia, Other or No Diagnosis). Pearson's chi‐square tests, Wilcoxon rank sum tests, and logistic regression models were used to evaluate associations between socioeconomic factors and health outcomes.

**Result:**

In the Eastern region, employment status was significantly associated with primary diagnosis (χ^2^ = 16.399, df = 4, *p* = 0.0025), while unemployment benefits were not (χ^2^ = 0.502, df = 1, *p* = 0.4785). Housing insecurity was significantly associated with cognitive health diagnosis (χ^2^ = 8.356, df = 1, *p* = 0.0038), with logistic regression indicating that stable housing reduced the odds of receiving a cognitive health diagnosis (β = ‐0.857, SE = 0.296, *p* = 0.0038). In the Western region, employment status was strongly associated with primary diagnosis (χ^2^ = 75.87, df = 4, *p* < 0.001), while unemployment benefits were not (χ^2^ = 0.06857, df = 1, *p* = 0.793). Housing stability significantly differed between cognitive health diagnosis groups (W=52544, *p* = 0.0001), with housing security also showing a strong association (χ^2^ = 8.3559, df = 1, *p* = 0.0038). Logistic regression revealed that unemployment, retirement, and not seeking work significantly increased the likelihood of a primary diagnosis (*p* < 0.001).

**Conclusion:**

Findings highlight employment and housing stability as key social determinants influencing cognitive health, emphasizing the need for policies that enhance economic security to mitigate dementia risk.

